# A pilot study to examine association of BMI with functional class and 6 min walk distance in idiopathic and heritable PAH: Possible association with estrogen metabolism

**DOI:** 10.1002/pul2.12139

**Published:** 2022-07-01

**Authors:** Margaret R. MacLean, Divya Pandya, Emilia M. Swietlik, Nina Denver, Kirsty Mair, Nicholas W. Morrell, Stefan Gräf, National Cohort Study for Idiopathic and Heritable Pulmonary Arterial Hypertension Consortium

**Affiliations:** ^1^ Institute of Pharmacy and Biomedical Sciences University of Strathclyde Glasgow UK; ^2^ Department of Medicine University of Cambridge Cambridge UK; ^3^ Department of Haematology University of Cambridge Cambridge UK; ^4^ NIHR BioResource for Translational Research University of Cambridge Cambridge UK

**Keywords:** estrogen, estrogen metabolites, obesity, pulmonary hypertension, sex

## Abstract

The hypothesis that a relationship exists between body mass index (BMI), functional class, and 6 min walk distance (6MWD) in Group 1‐pulmonary arterial hypertension (PAH) was examined. Analysis of data from the UK National Cohort Study for heritable pulmonary arterial/idiopathic PAH suggests increased BMI is a predictor of worse functional class and shorter 6MWD; increased body‐weight in mice and man may be associated with increased estrogen metabolism.

## INTRODUCTION

Some clinical studies suggest that obesity is associated with lower mortality in patients with precapillary pulmonary hypertension (PH).^1^, ^2^ However other studies on patients with a mixed etiology of pulmonary arterial hypertension (PAH) or PH secondary to sleep apnea, suggest more severe disease in obese patients or increased mortality among young patients who are morbidly obese.^3^, ^4^, ^5^ Weight loss, including bariatric surgery has been shown to improve pulmonary arterial pressures, symptoms, and exercise capacity in PAH patients.^6^ The relationship between body mass index (BMI) and severity of PAH in patients with Group 1 heritable PAH (HPAH) and idiopathic PAH (IPAH) is unclear. Here we investigated the association of BMI with PAH functional classification (according to the World Health Organization classification of functional status of patients with PH, 1998)^7^ and 6 min walk distance (6MWD) in IPAH and HPAH patients.

Blood estrogen levels are elevated in postmenopausal and male patients with iPAH.^8^, ^9^ It has been shown that diseased pulmonary arteries from male and female patients with PAH can synthesize estrogen via the enzyme aromatase and, unlike healthy pulmonary arteries, metabolize it via increased expression of the enzyme CYP1B1.^10^, ^11^ We have shown that this likely results in accumulation of estrogen metabolites in patients with IPAH and patients with porto‐pulmonary PAH.^12^, ^13^ Inhibition of aromatase or CYP1B1 can reverse PH in several animal models of PH including the sugen/hypoxic rodent model, the monocrotaline rat with PH, a dexfenfluramine‐induced mouse model, and transgenic mouse models^10^, ^11^, ^14^, ^15^, ^16^

We have previously shown that male ob/ob obese mice develop mild PH which is reduced by CYP1B1 inhibition. Consistent with this, there is also an increase in adipose tissue production of the mitogenic metabolite 16hydroxyestrone (16OHE1) which can induce proliferation and oxidative stress in hPASMCs and PH in mice.^11^, ^17^, ^18^ In human pulmonary arterial smooth muscle cells from PAH patients, estrogen can induce reactive oxygen species and this is abolished by CYP1B1 inhibition, suggesting estrogen may need to be metabolized to exert some of its pathogenic effects.^18^ Here we wished to analyse the relationship between BMI and 16OHEs in patients with PAH as well as body weight and urinary 16OHE1 in the male obese ob/ob mice.

The human data analysis was conducted on data from the UK National Cohort Study for HPAH and IPAH^19^ (release February 3, 2022) to examine the association between BMI, functional class (FC), and 6MWD. Data were drawn from a total of 1285 adult patients (69.1% females) diagnosed with IPAH (*n* = 1192) or HPAH (*BMPR2* mutation carriers, *n* = 93). Individuals with missing values were excluded. Patient characteristics are summarized in Table 1.

**Table 1:** Characteristics of the patients studied

Characteristic	HPAH (*N* = 93)^a^	IPAH (*N* = 1192)^a^	*p* Value
Age at diagnosis [years]	38 (30−52)	50 (38−65)	<0.001^b^
Sex			NS
Female *n* (%)	60 (65%)	828 (69%)	
Male *n* (%)	33 (35%)	364 (31%)	
BMI [kg/m^2^]	27 (23−33)	27 (23−32)	NS
(Missing)	1	107	
Functional class: number of patients			NS
1	3 (3.3%)	19 (1.6%)	
2	21 (23%)	218 (19%)	
3	58 (63%)	768 (66%)	
4	10 (11%)	152 (13%)	
(Missing)	1	35	
SMWD [m]	400 (289−472)	327 (207−413)	<0.005^b^
(Missing)	50	719	
RAP [mmHg]	8.0 (5.0−13.0)	8.0 (5.0−12.0)	NS
(Missing)	6	129	
mPAP [mmHg]	55 (48−65)	53 (44−61)	<0.05
(Missing)	3	66	
PAWP [mmHg]	9.0 (6.0−11.0)	9.0 (7.0−12.0)	NS
(Missing)	10	169	
PVR [mmHg/L*min]	14.7 (9.8−18.8)	11.0 (7.7−15.0)	<0.001^b^
(Missing)	14	211	

*Note*: Missing, *n*, patients where information not on database.

Abbreviations: BMI, body mass index; HPAH, heritable pulmonary arterial hypertension; IPAH, idiopathic PAH; mPAP, mean pulmonary arterial pressure; PAWP, pulmonary arterial wedge pressure; PVR, pulmonary vascular resistance; RAP, right atrial pressure; SMWD, six minute walk.

^a^ Median (interquartile range) age, BMI, SMWD, RAP, mPAP, PAWP, PVR. Wilcoxon rank sum test (age, sMWD, RAP, mPAP, PAWP, PVR).

^b^ Pearson's *χ* ^2^ test (sex). Fisher's exact test: functional class (functional class).

### BMI and 6MWD

**Figure 1:**
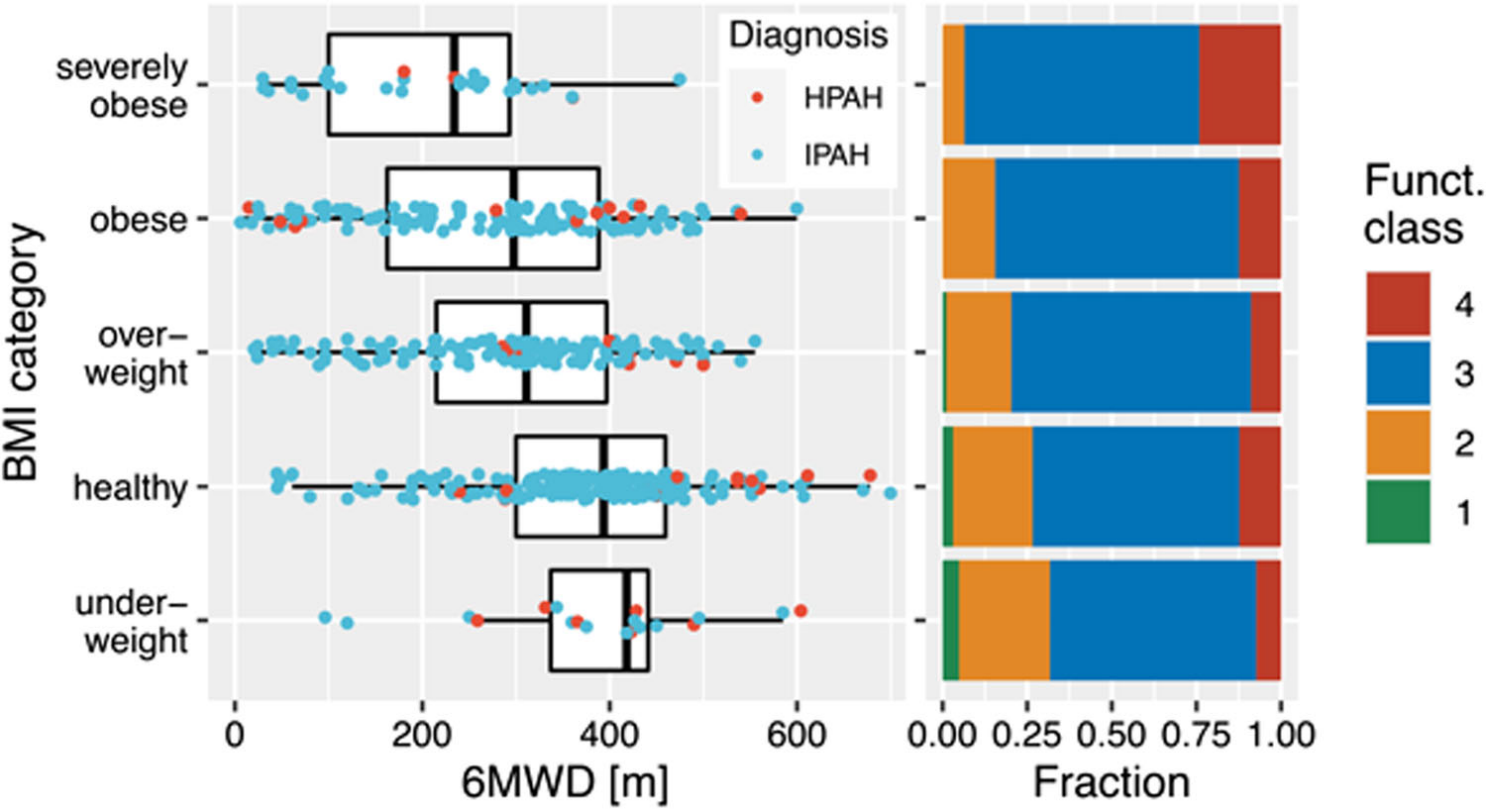
Distribution of (left hand panel) 6 min walk distance (6MWD) and (right hand panel) functional class (funct. class) stratified by body mass index (BMI) category (underweight: <18.5, healthy: ≥18.5 and <25; overweight: ≥25 and <30, obese: ≥30 and <40, severely obese: ≥40). 6MWD: heritable pulmonary arterial hypertension (HPAH, *n* = 43, 65% female); idiopathic PAH (IPAH, *n* = 460, 69% female). Funct. class: 1 HPAH, *n* = 3; IPAH, *n* = 19. 2 HPAH, *n* = 21; IPAH, *n* = 218. 3 HPAH, *n* = 58; IPAH, *n* = 768. 4 HPAH, *n* = 10; IPAH, *n* = 152.

The minimal linear additive model (6MWD ~BMI + sex + age at diagnosis) including 503 patients supports that BMI corrected for sex and age at diagnosis is a useful predictor of 6MWD with *p* = 2 × 10^−16^ (Figure 1 left hand panel); the higher the 6MWD, the lower the BMI (*p* = 3 × 10^−14^). The data has been assessed for normality using the standard diagnostic plots.

### BMI and FC

The association between BMI and WHO  FC was examined in 1149 individuals (69% female) using the nonparametric Kruskall−Wallis test. The Shapiro−Wilks test was used to determine whether the respective predictors were normally or non‐normally distributed. A Mann−Whitney test indicated that the median BMI differs significantly between FC 1 and 2 (*p* < 0.0025) and between FC 2 and 3 (*p* < 0.00031). The results suggest that BMI is good predictor for functional class (*p* < 0.001) (Figure 1 right hand panel). There was no significant effect of potential confounders such as sex or etiology of PAH.

### BMI in PAH patients and estrogen metabolites

Blood estrogen levels are increased in male and postmenopausal female IPAH patients.^8^, ^9^ We have also reported that 16OHE1 and 16hydroxyestradiol (16OHE2) levels are elevated in IPAH patients (at levels that cause cell proliferation)^12^, ^18^ and levels of 16OHE1 associate with severity of PAH.^12^ In IPAH patients, high 16OHE2 levels can be reduced, and the 6MWD increased following treatment with the estrogen receptor antagonist fulvestrant.^20^ We therefore looked at the association between BMI and plasma 16OHE2 levels in IPAH female patients. In this pilot study, increased 16OHE2 levels were associated with a higher BMI (*R* = 0.9, *p* = 0.004 [BMI 20−45, *n* = 8]).

### Obesity in mice and estrogen metabolites

In obese male ob/ob mice, using Pearson's (R) correlation coefficient (*n* = 12) we demonstrated that increased body weight was associated with increased urine levels of 16OHE1 (*p* < 0.001). We have also shown that obesity increases penetrance of PH in BMPR2^R899X^ mice and this may be related to increased estrogen metabolism in adipose tissue.^21^

Collectively, these pilot studies suggest that, in IPAH and HPAH patients, high BMI is associated with higher WHO functional class, lower 6MWD, and accumulation of 16OHE2. We have demonstrated that there is increased accumulation of mitogenic 16OHE1 and/or 16OHE2 in serum from PAH patients and patients with porto‐PH.^12^, ^13^ We have also previously shown that adipose tissue from obese mice can metabolize estrogen to 16OHE1 via CYP1B1 and that CYP1B1 inhibition in these mice inhibits PH.^17^ Here we have shown that there is increased urinary 16OHE1 with increased body weight in obese male ob/ob mice.

Collectively, these results suggest a relationship between obesity, severity of PAH, estrogen metabolism and, in mice, increase BMPR2 penetrance. Fully powered studies looking at obesity related effects of disease severity and estrogen metabolism are warranted.

## AUTHOR CONTRIBUTIONS

Divya Pandya, Emilia M. Swietlik, and Stefan Gräf collected and analyzed the clinical data. Margaret R. MacLean, Stefan Gräf, and Nicholas W. Morrell contributed to the planning, organization, and funding of the data analysis. Margaret R. MacLean conceptualized and organized the study. Kirsty Mair and Margaret R. MacLean conducted the metabolite analysis of the human plasma. Kirsty Mair and Margaret R. MacLean carried out the mouse studies.

## CONFLICT OF INTEREST

The authors declare no conflict of interest.

## ETHICS STATEMENT

Patients recruited to the study provided informed consent for genetic analysis and clinical data capture (REC REF: 13/EE/0325).
